# The long-term outcomes of a cohort of adolescents and adults from Greece with autism spectrum disorder

**DOI:** 10.1186/s12991-019-0250-6

**Published:** 2019-11-16

**Authors:** Isaia Sevaslidou, Christina Chatzidimitriou, Grigoris Abatzoglou

**Affiliations:** School of Medicine, Service of Child and Adolescent Psychiatry, AHEPA Hospital, Third Psychiatric Clinic, Aristotle University of Thessaloniki, Thessaloniki, Greece

**Keywords:** Autism, Autism spectrum disorders, Long-term outcome, Adult follow-up studies

## Abstract

**Background:**

Autism spectrum disorder (ASD) is a neurodevelopmental disorder. Although it is a lifelong condition, treatments and services can improve a person’s symptoms and ability to function. Research on the outcomes in adolescence and adult life and on the associated factors is limited. The objective of the present study is to examine the outcomes as well as the contributing factors in adolescents and adults diagnosed with ASD in Greece.

**Method:**

Participants included 69 parents of individuals diagnosed with ASD in their childhood. Interviews were conducted with the parents, and archived medical and psychological records were collected. Participants had been diagnosed in the Child and Adolescent Unit of the 3d Psychiatric Clinic of the AHEPA Hospital in Thessaloniki, Greece between 1990 and 2007.

**Results:**

The overall outcome was poor in most of cases (22.6% “very poor” and 24.5% “poor”); however, a substantial number had “good” (18.9%) or “very good” (22.6%) outcomes. Severity of initial diagnosis (*χ*^2^ = 65.956, DF = 8, *p* < 0.001), presence of comorbid disorders in childhood (*χ*^2^ = 14,085, DF = 4, *p* < 0.007), current comorbidity (*χ*^2^ = 15.834, DF = 4, *p* = 0.003), and certain developmental milestones [early acquisition of language skills (*χ*^2^ = 16.991, DF = 8, *p* = 0.030)] were positively correlated with adult outcomes.

**Conclusions:**

Overall outcomes in the Greek sample were consistent with international studies. It seems that important contributing factors are comorbidity and especially overall lower cognitive function (intellectual disability), but further research is needed as well as enhanced adult-oriented research and intervention programs.

## Background

Autism is a pervasive neurodevelopmental disorder characterized by serious deficits in verbal and non-verbal communication and social interaction as well as restricted patterns of behaviors and interests. Currently, the term “autism spectrum disorder” (ASD) (single category diagnosis) is employed in DSM-5 and will be used for the purposes of the present article.

Autism spectrum disorder is a developmental disorder, a lifelong condition affecting multiple aspects of a person’s life [1–3]. ASD has been extensively studied. However, comparatively fewer systematic studies have been conducted as to the developmental course and long-term outcomes and needs of individuals in adolescence and adult life [4, 5].

In general, overall outcomes for adults with ASD in terms of mental health, relationships, employment, and independent living are considerably poorer than for their same-age peers [5–11]. Steinhouse et al. [8], in one of their most recent and sophisticated meta-analyses, report that overall outcomes were remarkably impaired. Almost half of the participants (47.7%) showed poor or very poor outcomes in adult life, whereas 31.1% had fair outcomes and 19.7% good outcomes.

Findings also indicate that the majority display a varied pattern of improvement [12, 13]. According to a systematic review of longitudinal studies [14], there is a wide variability, both across and within studies, in cognitive outcomes, adaptive functioning, social integration, and independence.

Knowledge about factors that are associated with different outcomes is, however, limited, and the need for further studies is imperative [5, 8, 14]. Factors that appear to contribute to better outcomes in adolescence and adult life include higher intellectual and verbal functioning in childhood and lower autism symptom severity [5, 14–16]. Comorbidity at the time of the initial diagnosis, as well as during adolescence and adult life, is considered important [17]. The role of gender remains uncertain [5, 14].

The present research aims to study the outcomes of adolescents and adults with ASD within a Greek sample, as an extensive review of the literature revealed that no such research has been conducted in Greece. The research focuses on the overall outcomes (emotional, cognitive and social) of individuals with ASD as well as on the possible contributing factors.

## Materials and methods

### Materials

The sample consisted of 69 parents of individuals that had been diagnosed with ASD as children (32 mothers, 5 fathers and 16 couples of parents). They came from a pool of 106 parents of 53 individuals with ASD (22 adolescents and 31 adults). The mean age was 48.8 ± 6.2 years for the mothers and 53.3 ± 8.0 years for the fathers. All participants who were approached consented to participate and a written informed consent was obtained.

Diagnosis had been performed by the child psychiatric team of the hospital, during childhood and according to internationally valid diagnostic criteria, such as the Diagnostic and Statistical Manual of Mental Disorders (DSM) or the International Classification of Diseases (ICD). All past diagnosis were transcribed as DSM-5 diagnosis for reasons of homogeneity and to conform to the contemporary scientific literature.

All ASD individuals had been initially diagnosed in the Child and Adolescent Unit of the 3d Psychiatric Clinic of the AHEPA Hospital in Thessaloniki, Greece, between 1990 and 2007.

Data were collected from the parents during a follow-up evaluation of the individuals with ASD, by one of the authors as part of their Ph.D. research project. Information were collected on age and severity of childhood diagnosis, major developmental milestones (acquisition of independent walking ability, acquisition of speech, acquisition of bladder, and bowel control) and present outcomes in verbal functioning, educational level, social functioning, and autonomous and independent functioning (occupational status and independent living).

### Methods

The protocol included an interview with the parents concerning the complete history and current status of the patient. The interview focused on (1) gender, (2) severity at the time of the first diagnosis (as defined by the clinical specifiers of DSM-5, which describe the overall functioning of the individual), (3) comorbidities at the time of diagnosis, (4) comorbidities at the follow-up assessment, and (5) important developmental milestones: (a) age of language acquisition (acquisition of language before 5 years of age), (b) age of acquisition of independent walking ability (ability to walk independently at 1 year of age), and (c) age of acquisition of bowel and bladder control (before 5 years of age).

As there is not a universally accepted standardized measure of adult outcomes [18], this measure was developed by the authors, using internationally accepted outcome criteria [8] and based on an international classification created initially by Rutter [19] and advanced by other authors [3, 8, 13, 20].

Overall outcomes (OO) were classified in a six point ordinal measure ranging from “very poor” to “very good” (Table 1).

**Table 1:** Overall outcome classification

Overall outcome	Definition	*N*	%	Male (%)	Female (%)
Very poor	Severe deficits—totally unable to lead an independent existence	12	22.6	25.0	11
Poor	Marked deficits—requiring high levels of support and supervision	13	24.5	18.2	55.6
Fair	Some degree of independence but requiring support and supervision	6	11.3	11.4	11.1
Good	Capable of independent functioning but requiring some degree of support	10	18.9	22.7	0.0
Very good	High level of independence	12	22.6	22.7	22.2

This classification estimates the overall outcome by summing up points on various developmental domains leading to a composite score. The domains assessed for the adult participants are verbal functioning, educational level, social functioning, and independent functioning (occupational status and independent living). The domains assessed for the adolescent participants are verbal functioning, educational level, social functioning, and autonomous functioning [13, 21].

### Statistical analysis

All analyses were conducted using the Statistical Package for Social Sciences, version 25.0 (SPSS 25; IBM Corp., Armonk, NY, USA) for Windows. Descriptive statistics were computed for the study sample and frequency tables were determined.

Chi-square tests were used to analyze categorical variables in relationship with outcome categories. Categorical variables include gender, severity at the time of the initial (childhood) diagnosis, comorbidities at the time of the initial diagnosis, comorbidities at the follow-up assessment, important developmental milestones: (a) age of language acquisition (acquisition of language before 5 years of age), (b) age of acquisition of independent walking ability (ability to walk independently at 1 year of age), (c) age of acquisition of bowel and bladder control (before 5 years of age), and overall outcomes (OO) (Table 2).

**Table 2:** Chi-square tests between contributing factors and overall outcomes

	Outcome										
	Very poor		Poor		Fair		Good		Very good		*p*
	*N*	%	*N*	%	*N*	%	*N*	%	*N*	%	
Age stage											
Adolescents	0	0.0	9	40.9	3	13.6	4	18.2	6	27.3	0.010
Adults	12	38.7	4	12.9	3	9.7	6	19.4	6	19.4	
Sex											
Male	11	25.0	8	18.2	5	11.4	10	22.7	10	22.7	0.139
Female	1	11.1	5	55.6	1	11.1	0	0.0	2	22.2	
Age of acquisition of language											
Never	3	50.0	2	33.3	1	16.7	0	0.0	0	0.0	0.030
< 5 years	6	14.3	9	21.4	5	11.9	10	23.8	12	28.6	
> 5 years	3	100.0	0	0.0	0	0.0	0	0.0	0	0.0	
Age of acquisition of bowel and bladder control (years)											
< 3	4	18.2	5	22.7	1	4.5	4	18.2	8	36.4	0.373
> 3	8	28.6	7	25.0	4	14.3	5	17.9	4	14.3	

	*Μ*	SD	*Μ*	SD	*Μ*	SD	*Μ*	SD	*Μ*	SD	*p*
Age of acquisition of walking	14.8	7.1	14.0	3.6	13.8	3.5	13.9	2.9	12.8	1.4	0.822
Severity at initial assessment											
Support required	0	0.0%	0	0.0%	1	4.5%	9	40.9%	12	54.5%	< 0.001
Significant support required	1	9.1%	4	36.4%	5	45.5%	1	9.1%	0	0.0%	
Very significant support required	11	55.0%	9	45.0%	0	0.0%	0	0.0%	0	0.0%	
Comorbidity at initial assessment											
No	6	15.4%	7	17.9%	4	10.3%	10	25.6%	12	30.8%	0.007
Yes	6	42.9%	6	42.9%	2	14.3%	0	0.0%	0	0.0%	
Comorbidity today											
No	4	14.8%	4	14.8%	1	3.7%	7	25.9%	11	40.7%	0.003
Yes	8	30.8%	9	34.6%	5	19.2%	3	11.5%	1	3.8%	

The Kruskal–Wallis *H* test was used to detect possible significant differences in the mean age of onset of walking, based on the overall outcome of autistic individuals.

## Results

### A. Socio-demographic characteristics of participants—parents

The socio-demographic characteristics of the parents are summarized in Tables 3, 4, and 5.

**Table 3:** Socio-demographic characteristics of parents

Family structure	*N*	%
Married	39	73.6
Divorced	11	20.8
Widowed	3	5.7
Other children		
No	9	17.0
Yes	44	83.0

**Table 4:** Parent’s educational level

Parent’s educational level	*N*	%
Mother’s educational level		
University	17	32.1
Technical training	11	20.8
Secondary education	25	47.2
Father’s educational level		
Tertiary education	15	28.3
Technical training	5	9.4
High school	33	62.3

**Table 5:** Parent’s employment status

Employment status	*N*	%
Mother’s employment status		
Retired	4	7.5
Unemployed	14	26.4
Agricultural worker	2	3.8
Private employee	8	15.1
Liberal professions/freelance	10	18.9
Salaried	15	28.3
Father’s employment status		
Retired	9	17.0
Agricultural worker	5	9.4
Private employee	14	26.4
Freelance	17	32.1
Salaried	8	15.1

### B. Characteristics of the adolescents and the adults with autism spectrum disorders

The majority of the adolescents and adults with ASD were male (44 male and 9 female), in accordance with the international ASD prevalence rates. At the time of the study, the individuals were between 11 and 38 years old with the mean age being 19.4 ± 6.3 years. They were first diagnosed with ASD between 1.5 and 10 years of age. The mean age at the time of the diagnosis was 3.6 years. Severity of diagnosis was estimated at Level 1 (“requiring support”) for the majority of the participants (41.5%), at Level 2 (“requiring substantial support”) for 11 participants (20.8%) and Level 3 (“requiring very substantial support”) for 20 participants (37.7%). At the time of the first diagnosis, 39 participants did not present any comorbid disorders (73.6%), and 14 presented comorbid disorders (26.4%). Characteristics are summarized in Table 6.

**Table 6:** Characteristics of individuals with ASD

Sex	*N*	%
Male	44	83.0
Female	9	17.0
Age		
Age (mean ± SD)	19.415 ± 6.335	
Adolescents (11–18 years)	22	41.5
Adults (18+)	31	58.5

### C. Overall outcomes

Most of the participants had either very poor (22.6%) or poor outcomes (24.5%), and a substantial number had good (18.9%) or very good outcomes (22.6%). Overall outcomes are summarized in Table 1.

### D. Associations between overall outcomes and contributing factors

Chi-square test revealed no significance concerning gender (*χ*^2^ = 6.938, DF = 4, *p* = 0.139) and age appropriate acquisition of bladder and bowel control (*χ*^2^ = 4.252, DF = 4, *p* = 0.373).

On the contrary, it suggested the presence of a significant effect with (a) severity of the diagnosis in childhood (*χ*^2^ = 65.956, DF = 8, *p* < 0.001), (b) the presence of comorbid disorders in the initial diagnostic assessment in childhood (*χ*^2^ = 14.085, DF = 4, *p* < 0,007), (c) the presence of comorbid disorders at the follow-up assessment (*χ*^2^ = 15.834, DF = 4, *p* = 0.003), and (d) age of language acquisition (*χ*^2^ = 16.991, Β.Ε. = 8, *p* = 0.030).

The Kruskal–Wallis *H* test suggested no statistically significant difference in the mean age of onset of acquisition of independent walking skills based on the outcome of autistic individuals (*χ*^2^ = 0.913, DF = 3, *p* = 0.822).

In relation to gender, the percentage of male participants who presented a very poor outcome (25%) was higher than the percentage of female participants (11%).

Individuals who were evaluated at the first level of severity (“requiring support”) in the initial diagnostic assessment in childhood presented a good (40.9%)-to-very good outcome (54.5%), whereas those who were evaluated at the 3rd severity level (“requiring very substantial support”) presented a poor (45%)-to-very poor outcome (55%).

Most of the individuals with comorbid disorders diagnosed during childhood (mainly intellectual disability) showed a very poor (42.9%) to poor outcome (42.9%), and none presented a good or very good outcome (0%). Individuals with no comorbid disorder during childhood presented mostly a very good (30.8%)-to-good outcome (25.6%) and fewer presented poor (17.9%) and very poor (15.4%) outcomes.

Individuals with comorbid disorders at the follow-up evaluation showed a very poor (30.8%) or poor outcome (34.6%) and very few presented a good (11.5%) or very good outcome (3.8%).

Finally, outcome for individuals with late language acquisition (after 5 years of age) is mostly very poor (50.0%) or poor (33.3%), while individuals with early language acquisition (before 5 years of age) present mostly a good (23.8%) or very good outcome (28.6%).

## Discussion

This study showed that most of the participants had either very poor (22.6%) or poor outcomes (24.5%). These findings are consistent with the previous research reviews and meta-analysis which indicate that overall outcomes are generally poor in the majority of individuals with ASD in the terms of educational level, occupational status, and social and independent functioning [5, 8, 13, 20, 22–26].

However, a substantial number had good (18.9%) and very good outcomes (22.6%), which is consistent with studies that indicate that close to 20% of individuals with autistic disorders have a good outcome and around half encounter a poor or very poor long-term outcome [8].

There was no significant correlation between gender and outcome in agreement with the international literature. Most studies report that there is no significant impact of gender on autism symptoms, behavior, or social outcomes [5, 13, 27], although no definitive conclusions can be drawn as due to prevalence rates, there are very few female participants in most studies [14].

Severity of diagnosis was positively correlated with outcome. Severity in our study, as in many studies in the field, takes into account the overall functioning of the individual (social, communication, and behavioral impairments) [22]. Severity has been linked to reduced independence, limited social relationships, and lower rates of employment in adolescence and adulthood [5, 22, 27].

Comorbidity at the time of the initial diagnosis (childhood) as well as comorbidity at the follow-up assessment (adolescence and adulthood) emerged as contributing factors in accordance with the literature. Many studies provide evidence that behavioral deterioration was highest for individuals who presented comorbid disorders [20, 22, 28, 29]. This is especially true for individuals with intellectual disability, as many studies show that a lower IQ is correlated with increased behavioral problems as well as lower overall outcomes, whereas a higher IQ is correlated with better employment, social, and overall outcomes [22, 27, 28].

Acquisition of verbal skills before age 5 was positively correlated with better adolescent and adult outcome, which is supported by the previous studies and meta-analysis [5, 8, 22]. Early language skills are one of the most researched contributing factors and speech acquisition before age 5 or 6 as well as better childhood language and communication skills were consistently found to be important predictors of adult outcomes [14, 30, 31].

### Limitations

Certain methodological issues limit the generalizability of these findings.

Due to the small number of participants, it was not possible to use parametric statistics and to clarify the possible confounding effect of current comorbidity.

Another important limitation of this study, and of many studies in the field, is due to the participants’ inability to give their subjective point of view. Therefore, the study had to rely on other informants, namely the parents, to estimate outcomes.

As the study was based exclusively on a clinical population, the sample cannot be considered representative and certain groups may be underrepresented (high-functioning individuals with ASD and individuals from lower socio-economic groups).

Another limitation is that there are no standardized measurements for adult outcomes, and there is a need for defining and applying more rigorous and quantifiable outcome criteria.

## Conclusions

To our knowledge, this is the first research focused on long-term outcomes of individuals with ASD in Greece. Studies concerning adult outcomes should be a priority as individuals with ASD continue to face many challenges in adult life and specialist services and interventions remain comparatively weak [23].

In accordance with the literature, the overall outcome was very poor or poor in most cases as overall outcomes for adults with ASD in terms of relationships, employment, and independent living are considerably poorer in comparison with their same-age peers [5].

Severity of initial diagnosis, presence of comorbid disorders in childhood, current comorbidity, and early acquisition of language skills were positively correlated with adult outcomes. These findings suggest the need for additional improvement of early intervention and childhood treatment and interventions programs.

The findings of this study highlight the importance of designing and implementing long-term intervention programs for adolescents and adults with ASD focused on social integration and independent living. As many researchers note, whereas there is an increasing number of high-quality interventions for children, there are a few adult-centered intervention programs and very few studies on effective treatments and services for adults [26].

## Data Availability

The data that support the findings of this study are available from I.S., but restrictions apply to the availability of these data, which were used under license for the current study, and so are not publicly available. Data are, however, available from the authors upon reasonable request and with permission of I.S.

## Abbreviation

ASD: autism spectrum disorder
